# Limits to growth of forest biomass carbon sink under climate change

**DOI:** 10.1038/s41467-018-05132-5

**Published:** 2018-07-13

**Authors:** Kai Zhu, Jian Zhang, Shuli Niu, Chengjin Chu, Yiqi Luo

**Affiliations:** 1 Department of Environmental Studies, University of California, Santa Cruz, CA 95064 USA; 2 Tiantong National Station of Forest Ecosystem Research & Center for Global Change and Ecological Forecasting, School of Ecological and Environmental Sciences, East China Normal University, 200241 Shanghai, China; 3 Shanghai Institute of Pollution Control and Ecological Security, 200092 Shanghai, China; 4 Key Laboratory of Ecosystem Network Observation and Modeling, Institute of Geographic Sciences and Natural Resources Research, Chinese Academy of Sciences, 100101 Beijing, China; 5 University of Chinese Academy of Sciences, 100049 Beijing, China; 6 Department of Ecology, State Key Laboratory of Biocontrol & School of Life Sciences, Sun Yat-sen University, 510275 Guangzhou, China; 7 Center for Ecosystem Science and Society & Department of Biological Sciences, Northern Arizona University, Flagstaff, AZ 86011 USA

## Abstract

Widely recognized as a significant carbon sink, North American forests have experienced a history of recovery and are facing an uncertain future. This growing carbon sink is dictated by recovery from land-use change, with growth trajectory modified by environmental change. To address both processes, we compiled a forest inventory dataset from North America to quantify aboveground biomass growth with stand age across forest types and climate gradients. Here we show, the biomass grows from 90 Mg ha^–1^ (2000–2016) to 105 Mg ha^–1^ (2020 s), 128 Mg ha^–1^ (2050 s), and 146 Mg ha^–1^ (2080 s) under climate change scenarios with no further disturbances. Climate change modifies the forest recovery trajectory to some extent, but the overall growth is limited, showing signs of biomass saturation. The future (2080s) biomass will only sequester at most 22% more carbon than the current level. Given such a strong sink has limited growth potential, our ground-based analysis suggests policy changes to sustain the carbon sink.

## Abstract (web summary)

The recovery of North American forests is likely to impact their capacity as a carbon sink. Here, Zhu et al. show a growth in aboveground biomass in various climate change scenarios, with these forests expected to sequester no more than 22% more carbon than current levels by the 2080s.

## Introduction

North American forests have been widely recognized as a growing carbon sink, absorbing a substantial amount of CO_2_ from the atmosphere^1^. Two processes are commonly considered to dictate this growing carbon sink: forest recovery induced by land-use change or disturbances such as agricultural abandonment, reduced harvesting, and fire suppression; and growth trajectory modified by environmental changes such as CO_2_ fertilization, nitrogen deposition, and climate change^2^. These two growth processes could have contrasting implications to the fate of the carbon sink. If forest recovery is the dominant mechanism, then the current carbon sink is expected to saturate as forests age and reach late successional stages. In contrast, if modified growth dominates, then the carbon sink might continue to increase, offering additional potential for carbon sequestration. Previous studies have attributed the North American forest carbon sink to either regrowth, e.g., ref.^3^ or modified growth, e.g., ref.^4^, with limited consideration of both mechanisms. With rapid changes in land-use and climate in North America and around the world^5–7^, it is urgently important to have a full understanding of both contributing mechanisms and prediction of the potential of forest biomass as a carbon sink. This understanding can help inform policies on future CO_2_ emission targets and forest management strategies.

Here we compiled a complete set of forest inventory data from North America north of Mexico to understand the fate of forest biomass as a carbon sink and to predict its potential in mitigating climate change. The study of North American forests in depositing carbon is especially critical because they have experienced a history of resiliency and recovery: forests were mostly cleared for agriculture in the early twentieth century, but they since have significantly recovered^8^. The basic model of forest recovery postulates that biomass starts to accumulate with stand age as forests regrow following disturbance, but the trajectory is modified by changes in the environment. The aboveground biomass of a forest observed at a given age is an outcome integrated from both processes. To understand and predict this cumulative effect, we developed a hierarchical Bayesian (HB) growth model, assuming that the forest recovery trajectory is described by a growth function, where its parameters are further determined by forest-type and climate. We chose the Monod (Michaelis–Menten) growth function for its simplicity and similar performance among many growth functions (Supplementary Figures 1, 2). For each forest-type, the two Monod parameters, the asymptotic saturated aboveground biomass and the stand age to reach half-saturation, further depend on local climate conditions, with the assumption that the spatial variation in climate distribution can substitute the temporal variation in climate change^9^. Our model is verified and validated by the National Forest Inventory data of 26 yr (1990–2016) and 140,267 plots spanning across the United States and Canada. We began by developing the HB growth model using observations in the current period (2000–2016). We then used the current model to independently hindcast observations in the past period (1990–1999). Finally, we extrapolated our model to predict the forest biomass potential under idealized scenarios in the future periods (2020s, 2050s, 2080s) and quantify the extent to which the current biomass approaches the future biomass potential. We found that climate change effectively modifies the forest recovery trajectory, but the overall forest growth is limited. Under various climate change scenarios, the North American forest biomass will sequester at most 22% more carbon than current levels by the 2080s. The limits to forest growth suggest policy changes to lower future CO_2_ emission targets and actively manage forest resources.

## Results

### Forest recovery modified by climate

**Fig. 1:**
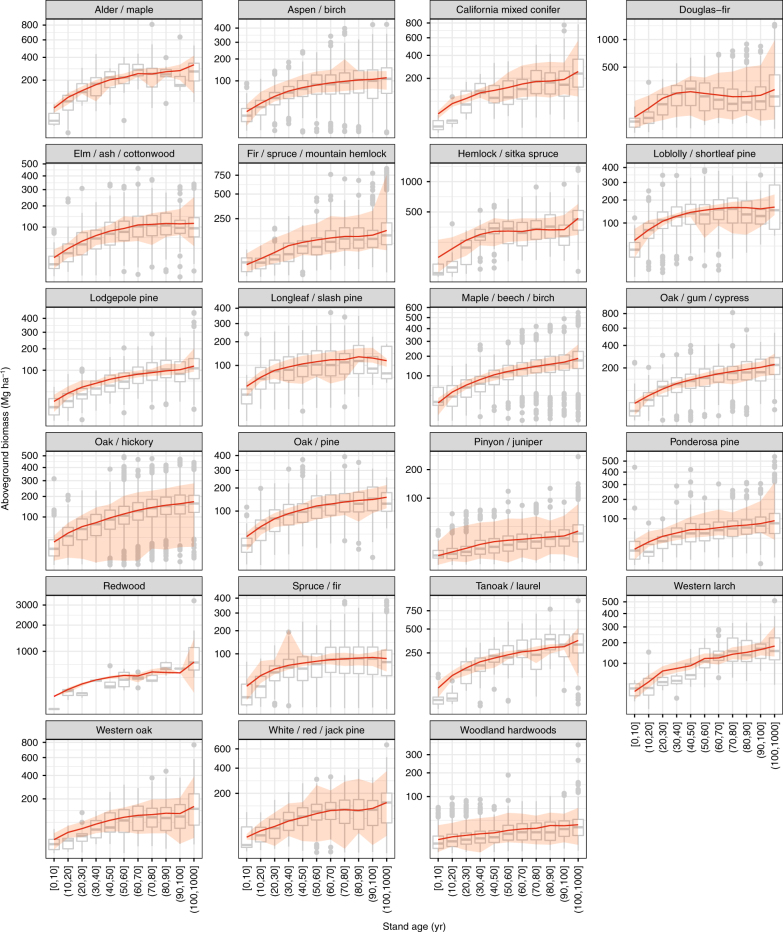
Forest aboveground biomass current recovery with stand age across forest-type. Observed and modeled aboveground biomass and stand age in 23 primary forest types across North America are summarized for the current period, 2000–2016. Observed values (gray boxplots) are collected from the National Forest Inventory programs in the United States and Canada. Modeled values (red lines and ribbons) are calculated as the posterior means (lines) and 95% credible intervals (ribbons) from the hierarchical Bayesian growth model fitted to the current data. Each forest-type is fitted to the model separately

**Fig. 2:**
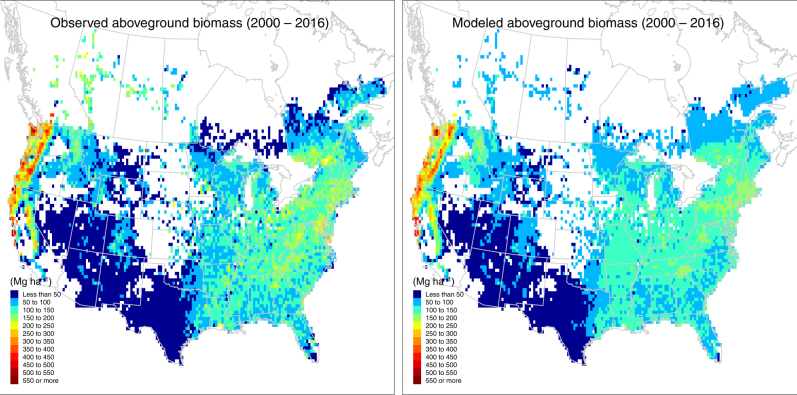
Geographic distributions of current forest aboveground biomass. Both the observed and modeled maps are averaged at the 10-min longitude by latitude resolution from inventory plots for the current period, 2000–2016. GIS data source: GADM database of Global Administrative Areas (https://gadm.org)

During the current period (2000–2016), our HB growth model successfully explains the large variations in the aboveground biomass across the stand age and geographic gradients of major forest types in North America. Figure 1 shows the measured aboveground biomass spanning 30-fold across the 1000 yr gradient of stand ages. Of the 23 forest types, western forests have significantly larger biomass than eastern forests (e.g., redwood vs. loblolly/shortleaf pine). In general, the aboveground biomass accumulates rapidly at young stand age and gradually saturates at later stages. The large variations within each age group and deviations from the monotonically increasing Monod function (e.g., decreasing biomass at some stages in Douglas-fir) suggest local climate effects in modifying the recovery trajectory. Comparing to observations, the model effectively quantifies the recovery trajectory and its uncertainty. The strong spatial correlation (*r* = 0.888, *p* < 0.001, spatially modified *t*-test) between the observed and modeled biomass indicates that the model predicts the biomass well (Fig. 2). Forests in the Pacific Northwest have the highest biomass density (>300 Mg ha^–1^) and the southwestern forests, in contrast, have the lowest biomass (<50 Mg ha^–1^), with the northeastern forests (150–250 Mg ha^–1^) and the southeast (100–200 Mg ha^–1^) falling between. Although data from the United States and Canada were retrieved from different sources, they show smooth transitions across the border. Agreements along the stand age gradient (Fig. 1) and across North American geography (Fig. 2) both verify the assumptions that biomass recovers with stand age, with trajectory modified by climate. We also performed a spatial cross-validation and found that the observed and predicted biomass are spatially highly correlated (*r* *=* 0.767, *p* *<* 0.001, spatially modified *t*-test), which provides yet another way to verify the assumptions.

The forest recovery trajectory is governed by two critical parameters: the asymptotic saturated aboveground biomass and the stand age to reach half-saturation, both of which are assumed to depend on climate. Across forest types, higher temperature generally increases the saturated aboveground biomass but decreases the half-saturation stand age; more abundant precipitation increases both the saturated biomass and half-saturation age. The estimated model parameters quantify the saturated biomass and half-saturation age on average climates, as well as various climate effects on them (Supplementary Table 1). Under the average temperature and precipitation across 23 forest types, the aboveground biomass saturates to 355 Mg ha^–1^ on average, ranging from 32.8 Mg ha^–1^ (woodland hardwoods) to 982 Mg ha^–1^ (redwood); the half-saturation age is 106 yr on average, ranging from 23.8 yr (longleaf/slash pine) to 252 yr (white/red/jack pine). For the saturated biomass, the temperature effects are 14 Mg ha^–1^ °C^–1^ on average, ranging from –65.2 Mg ha^–1^ °C^–1^ (oak/gum/cypress) to 144 Mg ha^–1^ °C^–1^ (redwood); the precipitation effects are 0.177 Mg ha^–1^ mm^–1^ on average, ranging from –0.440 Mg ha^–1^ mm^–1^ (white/red/jack pine) to 1.57 Mg ha^–1^ mm^–1^ (redwood). For the half-saturation age, the temperature effects are –1.20 yr °C^–1^ on average, ranging from –22.9 yr °C^–1^ (western larch) to 18.4 yr °C^–1^ (elm/ash/cottonwood); the precipitation effects are 0.00679 yr mm^–1^ on average, ranging from –0.306 yr mm^–1^ (white/red/jack pine) to 0.198 yr mm^–1^ (redwood). In summary, the local climate conditions effectively modify the biomass recovery trajectories. The recovery trajectory altered by environmental conditions as a moving attractor is a fundamental property of the transient dynamics of terrestrial carbon storage^10^.

As in many forest succession studies, our analysis is based on the approach assuming the variation in biomass over time (age) could be approximated by the variation across space, e.g., ref.^9^ The space-for-time assumption is widely applied but rarely tested. To validate this assumption, we used the model fitted to the current observations to independently hindcast (backward predict) the observed biomass in the past period (1990–1999). With a decade apart from the current period, the past period provides supplementary information on stand age distributions over forest successional dynamics (Supplementary Figure 3). The additional test shows that the past observed aboveground biomass is well hindcasted by the models across forest types (Supplementary Figure 4) and that the observed and hindcasted biomass are spatially strongly correlated (Supplementary Figure 5; *r* = 0.846, *p* < 0.001, spatially modified *t*-test). It validates that the local climate variations across space can approximate the forest recovery dynamics over time, at least to the decadal scales over which we are concerned.

### Limited forest growth potential

**Fig. 3:**
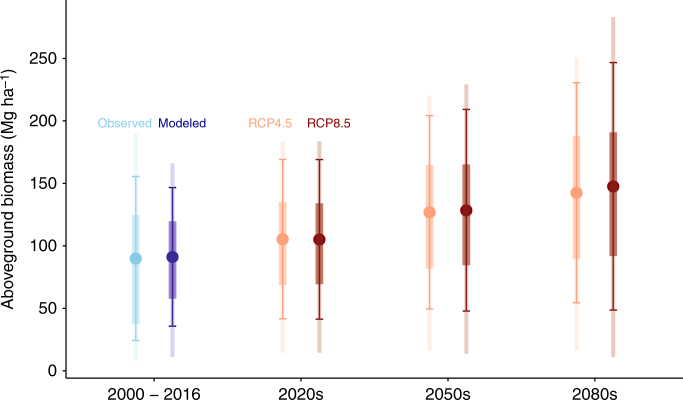
Trends of the current and future forest aboveground biomass. The current period (2000–2016) is summarized as observed and modeled values. The future periods (2020s, 2050s, 2080s) are summarized as two IPCC scenarios (RCP4.5 and RCP8.5) under the best-case circumstances of no disturbances. Points are means, error bars are standard deviations, thick lines are 50% quantiles (25% and 75%), and thin lines are 90% quantiles (5% and 95%)

**Fig. 4:**
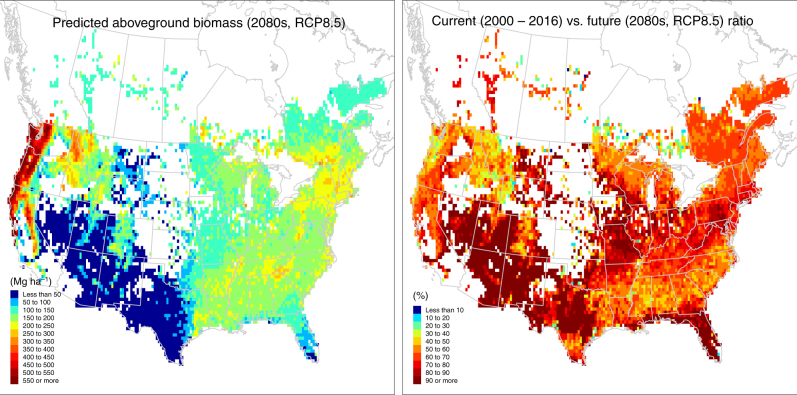
Geographic distributions of future forest aboveground biomass and current vs. future ratio. The modeled aboveground biomass across North America during the future period, 2080s, under the RCP8.5 scenario, is used together with the current biomass (Fig. 2) to calculate the ratio of current vs. future biomass. The ratio summarizes the extent to which the current biomass approaches the future biomass potential under the best-case circumstances of no disturbances. Both maps are averaged at the 10-min longitude by latitude resolution from inventory plots. GIS data source: GADM database of Global Administrative Areas (https://gadm.org)

Based on the validated results for current and past observations, we extrapolated the model of aboveground biomass into the future periods (2020s, 2050s, 2080s). As a starting point to anticipate climate change, we aimed to quantify the potential of biomass carbon sequestration under best-case scenarios. With this goal, we assumed no major shift in forest-type composition and no further disturbance, such as fire or insect outbreak. We considered the projected change in climate, because they could modify forest recovery trajectories. Figure 3 shows the current period (observed and modeled), in reference to the future periods under two climate change scenarios (low emission RCP4.5 and high emission RCP8.5). Biomass increases from the observed 89.9 ± 65.6 Mg ha^–1^ (mean ± standard deviation) or modeled 91.2 ± 55.5 Mg ha^–1^ in 2000–2016 (current), to 105 ± 63.8 Mg ha^–1^ (RCP4.5) or 105 ± 63.9 Mg ha^–1^ (RCP8.5) in the 2020s, to 127 ± 77.4 Mg ha^–1^ (RCP4.5) or 129 ± 80.7 Mg ha^–1^ (RCP8.5) in the 2050s, to 143 ± 88.1 Mg ha^–1^ (RCP4.5) or 148 ± 99 Mg ha^–1^ (RCP8.5) in the 2080s. Geographically, the biomass accumulation is dominated by the continued recovery of the eastern forests, supplemented by the northwestern forests (Supplementary Figure 6). Figure 4 shows an example of the highest projection under RCP8.5 in the 2080s.

In addition, we calculated a ratio of the current vs. future modeled biomass in a geographic context, which summarizes the extent of current biomass approaching the future biomass under the best-case scenario (no disturbance). As an example, this ratio is 0.780 ± 0.438 for the current vs. the 2080s RCP8.5 future (Fig. 4), that is, the current forest biomass is on average 78% relative to the future biomass for the 2080s under the RCP8.5 best-case scenario. Because of the no-disturbance assumption, the actual future biomass is likely to be lower, and the actual ratio is likely to be higher, indicating that the biomass is likely to be even more saturated than 78%. In other words, under the unlikely best circumstances of no disturbances, North American forest carbon will only increase at most 22% over the current level to the 2080s.

## Discussion

Our findings suggest that North American forests are recovering from previous disturbances with trajectories modified by climate, but the biomass growth potential appears limited. Although the limited growth of forest biomass with age is known in theory^11^, this study uses ground-based measurements to quantify the extent under idealized projected climate change scenarios across continental scales. Verified by the current data and validated by the past observations, our model predicts that the present aboveground biomass is, averaged across all forest types, 78% of its biological capacity in the 2080s future of North America. The future projection assumes the best-case scenario as no further disturbance will reset the successional clock. Clearly, the actual future biomass is likely to be lower than this ideal projection, as additional disturbances reset the ecosystem to earlier successional stages. Given the increasing intensity of disturbances under global change^5–7^, our projections of the biomass ratio are likely to be underestimated. In other words, increased future disturbances might lead to an even smaller biomass carbon sink and a higher ratio than reported here. All these signs point to limits to forest growth and saturation of forest biomass carbon sink across North America. These findings are consistent with other observation-based models in the United States^12^ and echoes similar findings in Europe^13^. Because such a strong sink of North American forest biomass (offset > 10% of current CO_2_ emissions)^14^ is shown to be limited, future CO_2_ emission targets might need to be lowered.

Our analysis comes with several possible limitations. First, it does not consider environmental change factors other than climate (temperature and precipitation). Changes in atmospheric composition, such as CO_2_ and nitrogen, have been shown to enhance forest growth^15,16^. On the one hand, our past and current periods (1990–2016) might not be sufficiently long to detect the CO_2_ and nitrogen fertilization effects. On the other hand, our future periods (2020s, 2050s, 2080s) might have different levels of CO_2_ concentration and nitrogen deposition than today, and they could affect forest dynamics and biomass. The potential impacts of CO_2_ and nitrogen fertilization are knowledge gaps that worth further investigation. Another possible factor is growing season length, which might have considerable positive influence on forest growth. Seasonal forest ecosystems might not be sensitive to temperature or precipitation per se, but they might respond to the prolonged growing season, as suggested in studies of accelerated growth^4,17^.

Second, our study could miss the influences of future land-use changes on forest biomass, such as afforestation and deforestation from urban growth, conversion to other types by agriculture practices, and woody encroachment into grasslands. All these changes might affect forest carbon sequestration potential as forests grow and recover. However, extensive studies have shown forest migration is much slower than expected, with particular concern of “migration lag” for plants^18,19^. Plant movements have not yet been realistically represented in models used to predict future vegetation and carbon-cycle feedbacks^19^. Here we took the first step to quantify biomass carbon potential under the best-case circumstances with no shifts in forest geographic distributions. We anticipate the quantification to improve with the progress in understanding forest distributional responses to global change.

Finally, our analysis does not include the belowground components due to data limitation. Belowground carbon pools (e.g., root and soil carbon) have been shown to have different recovery trajectories^20,21^ and responses to climate change^22^ compared with aboveground components. Therefore, our results should be explicitly limited to the aboveground biomass carbon.

Based on in-situ observations, our analysis suggests that North America forests, after decades of increasing biomass carbon, might start to experience growth saturation, and their carbon sequestration potential is limited at 22% to the 2080s future. The saturation of forest biomass has been suggested by simulation models^23,24^, and now it is substantiated by our analysis of ground measurements. Our results also demonstrate the importance of considering forest age profiles^4,25,26^ and disturbance regime shifts under global change^27^. Putting into a broader context, the limited forest growth is coupled with increased mortality^28–30^ as well as slow migration^18,19^. All these stresses call for programs to actively manage our vital forest resources in an ever-changing global environment^7^.

## Methods

### Forest inventory and climate data

We assembled forest inventory data from the United States Forest Inventory and Analysis (FIA) program and the Canadian Permanent Sample Plots (PSP) program in six provinces (British Columbia, Alberta, Saskatchewan, Manitoba, Ontario, Quebec). The FIA program applies a nationally standardized sampling protocol with a sampling intensity of one plot per 2428 ha^31^. FIA inventory plots in forested areas consist of four 7.2 m fixed-radius subplots spaced 36.6 m apart in a triangular arrangement with one subplot in the center. All trees (standing live and dead), with a diameter at breast height (DBH) of at least 12.7 cm, are inventoried in each subplot. Within each subplot, a 2.07 m radius microplot offset 3.66 m from subplot center is further established where only live trees with a DBH between 2.5 and 12.7 cm are inventoried. All stems are measured and identified to species. For each plot, the age is determined by coring three dominant or co-dominant trees that represent a plurality of non-overtopped trees. The stand age is estimated as the average of these three trees^31^. The PSP program applies slightly different field protocols with FIA, with varying plot size and minimum measured DBH in six provinces^32^. Each PSP plot was designed as one square or rectangular plot in shape, or consist of four squared subplots. The average PSP plot size is 0.07 ha, ranging from 0.04 to 0.81 ha. All live trees with DBH > 1 cm were measured for most PSP plots, and the live trees with DBH > 5 cm were measured for a small group of PSP plots.

In this analysis, the FIA and PSP data were extracted from two periods: 2000–2016 (current) and 1990–1999 (past). We used the current period to develop a forest biomass–age relationship and the past period to independently validate this relationship. We excluded plots that reported any natural or human-caused disturbances, such as fire, logging. For the current period, we collected 67,065 FIA plots and 10,342 PSP plots. For the past period, we collected 54,127 FIA plots and 8733 PSP plots. The stand age was directly reported from plot estimates of coring trees. Our assumption was not that forest stands across North America are even-aged; rather we assumed that the age of the dominant or co-dominant trees represents the age of the forest ecosystem. For each plot, the aboveground biomass was estimated using allometric equations from DBH measurements^33^ and was summed across all live trees to obtain a live-tree plot biomass^34^. We classified these plots into 23 common forest types in North America. For the FIA plots, the forest types were included in the raw data, as derived from the dominant species to reflect the main species composition^35^. For the PSP plots, we classified the forest types following a similar approach by assigning the first 1–3 species with the highest percentage in aboveground biomass as the dominant species for each plot, and then we matched the forest types with the FIA classification.

Climate data in this study were extracted from ClimateNA version 5.10^36^. For forest inventory plots in both the past and current periods, we extracted the mean annual temperature and precipitation resampled at 1 km resolution using PRISM data^37^ from ClimateNA. For the future period, we extracted the downscaled (1 km) and calibrated (bias corrected) mean annual temperature and precipitation using the Coupled Model Intercomparison Project phase 5 (CMIP5) database corresponding to the 5th IPCC Assessment Report^38^. We used the ensemble projections averaged across the 15 CMIP5 models, under the Representative Concentration Pathway (RCP) 4.5 (low emission) and RCP8.5 (high emission) scenarios, for three future periods: the 2020s, 2050s, and 2080s. We acknowledged that the FIA plot coordinates have been perturbed in an unbiased direction not exceeding 1.67 km, and typically within a 0.8 km radius of the actual plot location, so as to facilitate study repeatability without introducing bias^39^. However, this perturbation is similar to the spatial resolution of our climate data (1 km). We therefore used the publicly available perturbed plot coordinates to match the forest inventory with climate data.

### Growth model selection

Prior to formal modeling, we performed an exploratory data analysis to select the most appropriate growth model. A growth model quantifies how forest biomass changes with stand age,

1 $$\begin{array}{*{20}{c}} {y_i = f\left( {x_i} \right) + {\it{\epsilon }}_i} \end{array}$$

where for plot *i*, *y*_*i*_ is the aboveground biomass, *x*_*i*_ is the stand age, and *ε*_*i*_ is the error term. In theory, a growth model has to go through the origin for the biomass–age relationship, i.e., $f\left( 0 \right) = 0$. Thus, we tested the following four commonly used growth models. Linear growth model, where the biomass increases with the age linearly.

2 $$\begin{array}{*{20}{c}} {f\left( {x_i} \right) = \beta x_i} \end{array}$$

Exponential growth model, where the biomass is a function of the natural-logarithm-transformed age^9^.

3 $$\begin{array}{*{20}{c}} {f\left( {x_i} \right) = \beta \log \left( {x_i + 1} \right)} \end{array}$$

Chapman–Richards growth model, where the biomass increases with the age and reaches an asymptote^40^.

4 $$\begin{array}{*{20}{c}} {f\left( {x_i} \right) = \mu \left( {1 - \exp \left( { - kx_i} \right)} \right)} \end{array}$$

Monod (Michaelis–Menten) growth model, where the biomass also increases with the age and reaches an asymptote^4^.

5 $$\begin{array}{*{20}{c}} {f\left( {x_i} \right) = \mu \frac{{x_i}}{{k + x_i}}} \end{array}$$

We fitted these four growth models to the data by different forest types (Supplementary Figure 1) and calculated their Akaike information criterion (AIC) scores. AIC measures the relative quality of models for a given set of data, by estimating the information lost when the model represents the process that generates the data. Practically, AIC rewards goodness of fit (likelihood) and penalizes overfitting (number of parameters). A lower AIC indicates a preferred model. Supplementary Figure 2 shows that, by AIC scores, the non-saturating models (linear and exponential) are outcompeted by the saturating models (Chapman–Richards and Monod). The Chapman–Richards model and Monod (Michaelis–Menten) model have similar AIC scores. Empirically, the Monod model growth trajectory is determined by two parameters with simple and clear definitions—the asymptote for the saturated biomass (*μ*) and the age when the plot reaches the half-saturation (*k*). We therefore chose the Monod model for similar performance and simplicity.

### Hierarchical Bayesian growth model

Our analysis quantifies the forest biomass recovery with stand age, with considerations of climate, in the past, current, and future forests across North America, in a hierarchical Bayesian (HB) framework. Theoretically, the HB framework was motivated by the transient dynamics of terrestrial carbon storage^10^. In a steady state environment, a growth function mathematically describes forest recovery in an autonomous system. When the environmental conditions are undergoing changes, the carbon storage capacity is no longer a constant but becomes a moving attractor, toward which the ecosystem carbon storage trajectory chases. In this non-steady state environment, a hierarchical growth model describes modified forest recovery in a non-autonomous system. A similar HB framework was recently developed to quantify climatic controls of postfire plant regeneration in South Africa^41^.

Our road map can be divided into three steps. First, we developed a HB growth model of the forest biomass, stand age, and climate relationship based on forest inventory and climate data in the current period (2000–2016). For each plot, the biomass recovery with stand age was effectively described by a Monod function, where the biomass gradually increases with the age but eventually saturates^4^. The Monod recovery trajectory is further determined by forest types and modified by climate conditions^10^. Second, we validated our model by using the current period to independently hindcast (backward predict) forest biomass in the past period (1990–1999). The model parameters obtained from the current period were used to hindcast the past biomass based on the past stand age and climate. The comparison of hindcast biomass vs. observed biomass provided an additional test of model performance and assumptions. Third, we used the validated model, future stand age, and future climate to predict forest biomass in the future periods of the 2020s, 2050s, and 2080s. We assumed no major disturbances as the best-case scenario for biomass recovery. Our final step was to compare the current biomass with the predicted future biomass. With this road map, we describe our analysis for the current, past, and future periods.

Current period—For the forest plot *i* in forest-type *j*, we modeled the aboveground biomass (*y*_*ij*_) and stand age (*x*_*ij*_) using the Monod function,

6 $$\begin{array}{*{20}{c}} {y_{ij} = \mu _{ij}\frac{{x_{ij}}}{{k_{ij} + x_{ij}}} + {\it{\epsilon }}_{ij},{\it{\epsilon }}_{ij}\sim N\left( {0,\sigma _j^2} \right)} \end{array}$$

where *μ*_*ij*_ is the asymptote for the saturated biomass that a plot can achieve, *k*_*ij*_ is the age when the plot reaches the half-saturation (*μ*_*ij*_/2), and *ε*_*ij*_ is the normal error term with a forest-type-level variance. The Monod function assumes that the forests recover by increasing biomass with stand age, but eventually reach an asymptote of the biological capacity, as suggested by both forest succession theory^11^ and empirical global meta-analysis^42^. The Monod recovery trajectory is governed by the two critical parameters (*μ*_*ij*_, *k*_*ij*_), which are assumed to further depend on the climate covariates of temperature (*T*_*ij*_) and precipitation (*P*_*ij*_) in each plot, motivated by the model of climate dependence in biomass accumulation rates^43^. The climate covariates (*T*_*ij*_, *P*_*ij*_) were centered to facilitate interpretation.

7 $$\begin{array}{*{20}{c}} {\mu _{ij} = \beta _{0j} + \beta _{1j}\left( {T_{ij} - \bar T_j} \right) + \beta _{2j}\left( {P_{ij} - \bar P_j} \right)} \end{array}$$

8 $$\begin{array}{*{20}{c}} {k_{ij} = \gamma _{0j} + \gamma _{1j}\left( {T_{ij} - \bar T_j} \right) + \gamma _{2j}\left( {P_{ij} - \bar P_j} \right)} \end{array}$$

where *β*_0*j*_ quantifies the asymptotic saturated biomass on an average climate condition, *β*_1*j*_ quantifies the saturated biomass change per 1 °C change in temperature, with $\bar T_j$ being the average temperature in forest-type *j*, and *β*_2*j*_ quantifies the saturated biomass change per 1 mm change in precipitation, with $\bar P_j$ being the average precipitation in forest-type *j*; *γ*_0*j*_ quantifies the half-saturation stand age on an average climate condition, *γ*_1*j*_ quantifies the half-saturation age change per 1 °C change in temperature, and *γ*_2*j*_ quantifies the half-saturation age change per 1 mm change in precipitation. All parameters were assigned priors sufficiently noninformative, so that the posterior estimates were driven by the observed data.

9 $$\begin{array}{*{20}{c}} {\beta _{0j},\beta _{1j},\beta _{2j},\gamma _{0j},\gamma _{1j},\gamma _{2j}\sim U\left( { - 10^5,10^5} \right)} \end{array}$$

10 $$\begin{array}{*{20}{c}} {\sigma _j^2\sim \mathrm{IG}\left( {10^{ - 5},10^{ - 5}} \right)} \end{array}$$

where *U* is the uniform distribution, and *IG* is the inverse gamma distribution. We also tested weakly informative priors on $\beta _{0j},\gamma _{0j}\sim U(0,10^5)$, implying the positive asymptotic saturated biomass and half-saturation stand age on an average climate condition, and we obtained almost identical posterior estimates. The model performance was checked using in-sample predictions by composite sampling. As another way of model checking, we performed a spatial cross-validation by randomly selecting 75% of the plots as the training dataset and 25% of the plots as the testing dataset. We fitted the model using the training plots, predicted biomass in the testing plots, and compared against the observed biomass. A good agreement between the out-of-sample predicted and observed biomass would verify the model assumptions and performance.

Past period—The model fitted using the current data assumes that the recovery trajectory in each plot differs by the spatial variation in climate, which can substitute the temporal variation in climate over the recovery e.g., ref.^9^ To validate this space-for-time assumption, we conducted an independent hindcast of the past biomass based on the stand age, climate, and fitted parameters from the current model. Plot distributions of both the past and current periods also show the dynamics of forest succession (Supplementary Figure 3). To conduct the test, for each plot *i* in forest-type *j*, the observed stand age in the past period (*x*_*ij*_), we obtained out-of-sample predictions of biomass ($\hat y_{ij}$), which is independent of the observed biomass in the past period.

11 $$\begin{array}{*{20}{c}} {\hat y_{ij} = \hat \mu _{ij}\frac{{x_{ij}}}{{\hat k_{ij} + x_{ij}}}} \end{array}$$

12 $$\begin{array}{*{20}{c}} {\hat \mu _{ij} = \hat \beta _{0j} + \hat \beta _{1j}\left( {T_{ij} - \bar T_j} \right) + \hat \beta _{2j}\left( {P_{ij} - \bar P_j} \right)} \end{array}$$

13 $$\begin{array}{*{20}{c}} {\hat k_{ij} = \hat \gamma _{0j} + \hat \gamma _{1j}\left( {T_{ij} - \bar T_j} \right) + \hat \gamma _{2j}\left( {P_{ij} - \bar P_j} \right)} \end{array}$$

where $\hat \beta$’s and $\hat \gamma$’s are fitted parameters from the current model. The model hindcast biomass ($\hat y_{ij}$) was first calculated from Eqs. (11–13) and then compared against the observed biomass. A good agreement between the independent hindcast and the observed biomass would suggest the current model performed well under the assumptions.

Future period—We assumed that the future North American forests continue to recover without major disturbances (e.g., fire, pest). This simplified assumption led to the best-case scenario of forest recovery, and it was likely to over-predict the future biomass. Our goal was not to accurately project the future biomass; rather we aimed to quantify how much the forest biomass can grow (biomass potential) given no further disturbances as the best-case scenario. With the validated model, we predicted the future biomass ($\tilde y_{ij}$) based on the future climate and stand age ($\tilde x_{ij}$).

14 $$\begin{array}{*{20}{c}} {\tilde y_{ij} = \tilde \mu _{ij}\frac{{\tilde x_{ij}}}{{\tilde k_{ij} + \tilde x_{ij}}}} \end{array}$$

15 $$\begin{array}{*{20}{c}} {\tilde \mu _{ij} = \hat \beta _{0j} + \hat \beta _{1j}\left( {\tilde T_{ij} - \bar T_j} \right) + \hat \beta _{2j}\left( {\tilde P_{ij} - \bar P_j} \right)} \end{array}$$

16 $$\begin{array}{*{20}{c}} {\tilde k_{ij} = \hat \gamma _{0j} + \hat \gamma _{1j}\left( {\tilde T_{ij} - \bar T_j} \right) + \hat \gamma _{2j}\left( {\tilde P_{ij} - \bar P_j} \right)} \end{array}$$

where $\tilde T_{ij}$ and $\tilde P_{ij}$ are the projected mean annual temperature and precipitation in the 2020s, 2050s, or 2080s. With no further disturbances, the future stand age was extrapolated from the current stand age extending to the future. The predicted future biomass $\left( {\tilde y_{ij}} \right)$ was compared with the current biomass $\left( {y_{ij}} \right)$. A ratio of current vs. future biomass $\left( {y_{ij}/\tilde y_{ij}} \right)$ is defined to quantify the forest recovery in reference to the best-case biomass potential. A ratio close to one would suggest that the forests have limited remaining growth potential. Note that the actual ratio is likely to be higher given further disturbances would result in lower future forest biomass.

For the HB growth model, posterior distributions were simulated using Markov chain Monte Carlo (MCMC), and convergence was checked by both visually assessing trace plots and Geweke diagnostics after 100,000 iterations for five Markov chains. All the analyses were performed in R version 3.4.3^44^ and JAGS version 4.3.0^45^.

### Data availability

The United States forest inventory data are available at https://www.fia.fs.fed.us/. The Canadian forest inventory data are available from forestry sectors in each province. The climate data are available at https://adaptwest.databasin.org/pages/adaptwest-climatena.

### Code availability

The code used in this study is available from the corresponding author on request.

## Electronic supplementary material

Supplementary Information
