# Supplementary material for: Limits to growth of forest biomass carbon sink under climate change
Source: Nat Commun. 2018 Jul 13;9:2709. doi: 10.1038/s41467-018-05132-5 (PMC6045605; doi:10.1038/s41467-018-05132-5)
Supplement: Supplementary file 1 — Supplementary Information [file 41467_2018_5132_MOESM1_ESM.pdf]

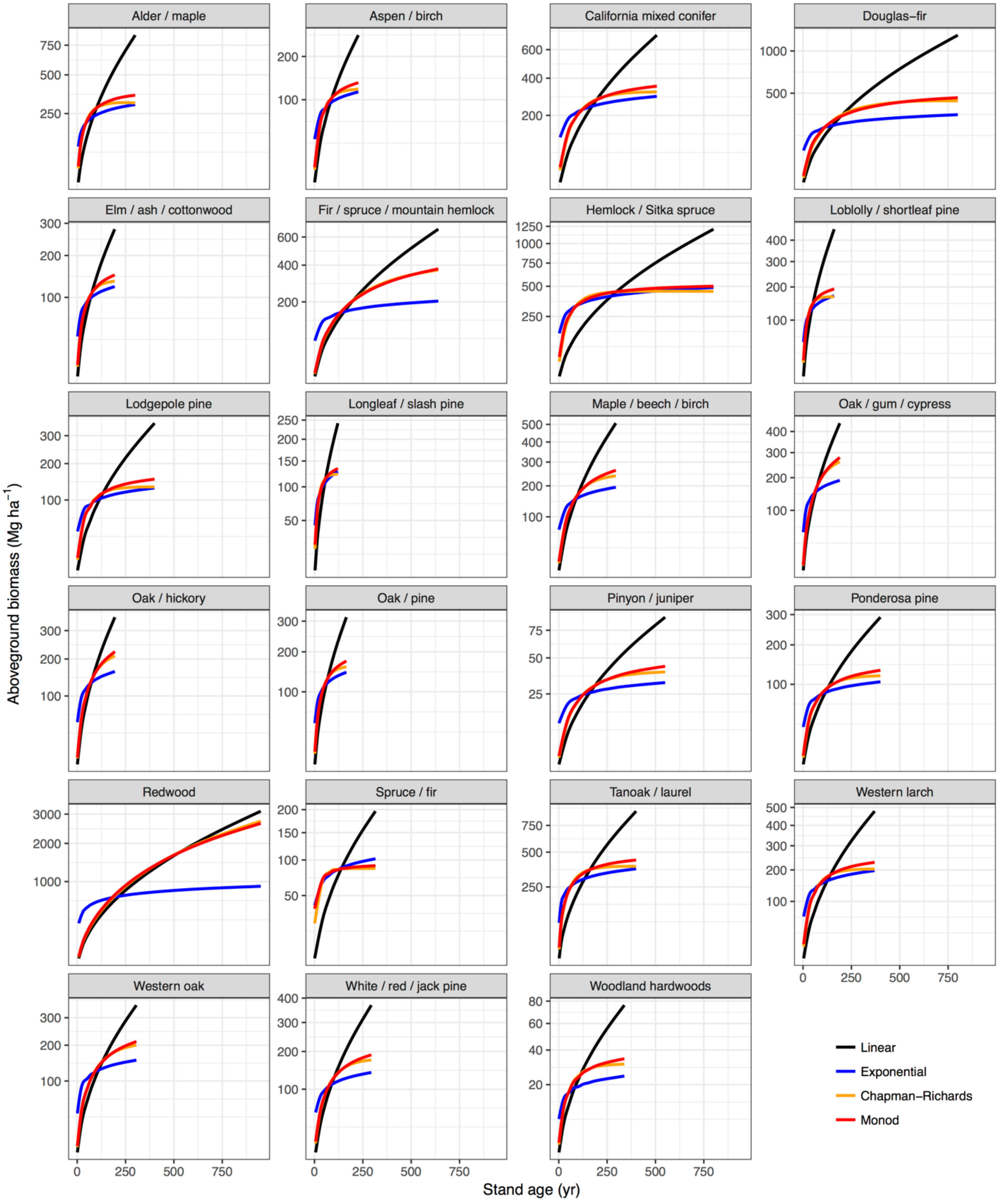

Supplementary Figure 1 Comparison of growth models by fit. Models are fitted to the aboveground biomass and stand age across forest-type data. The four candidates are the linear, exponential, Chapman-Richards, and Monod (Michaelis-Menten) growth models. Note the vertical axes are on square-root scales.

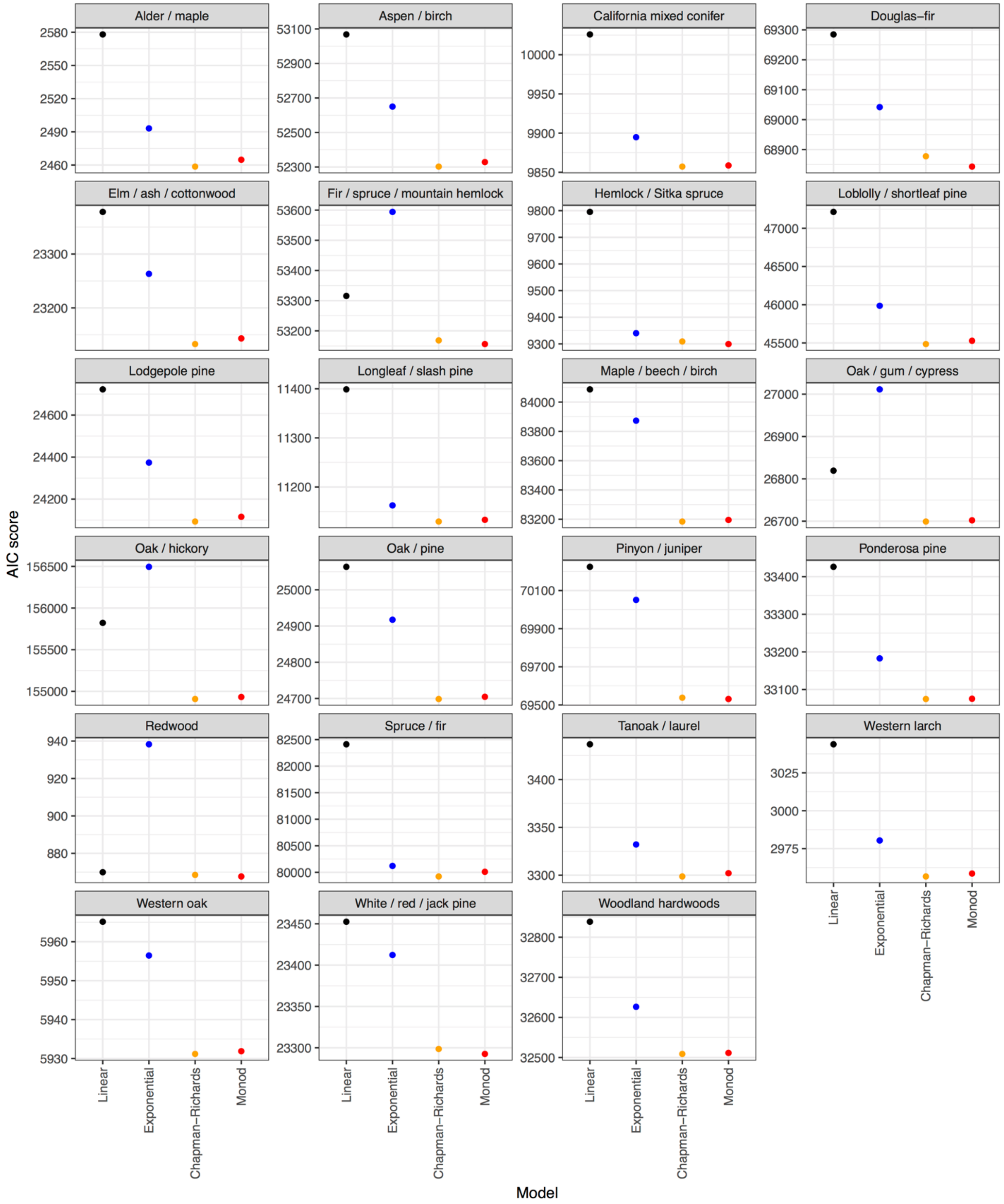

Supplementary Figure 2 Comparison of growth models by AIC. The four candidate models are compared by the Akaike information criterion (AIC) scores. Symbols follow Supplementary Figure 1.

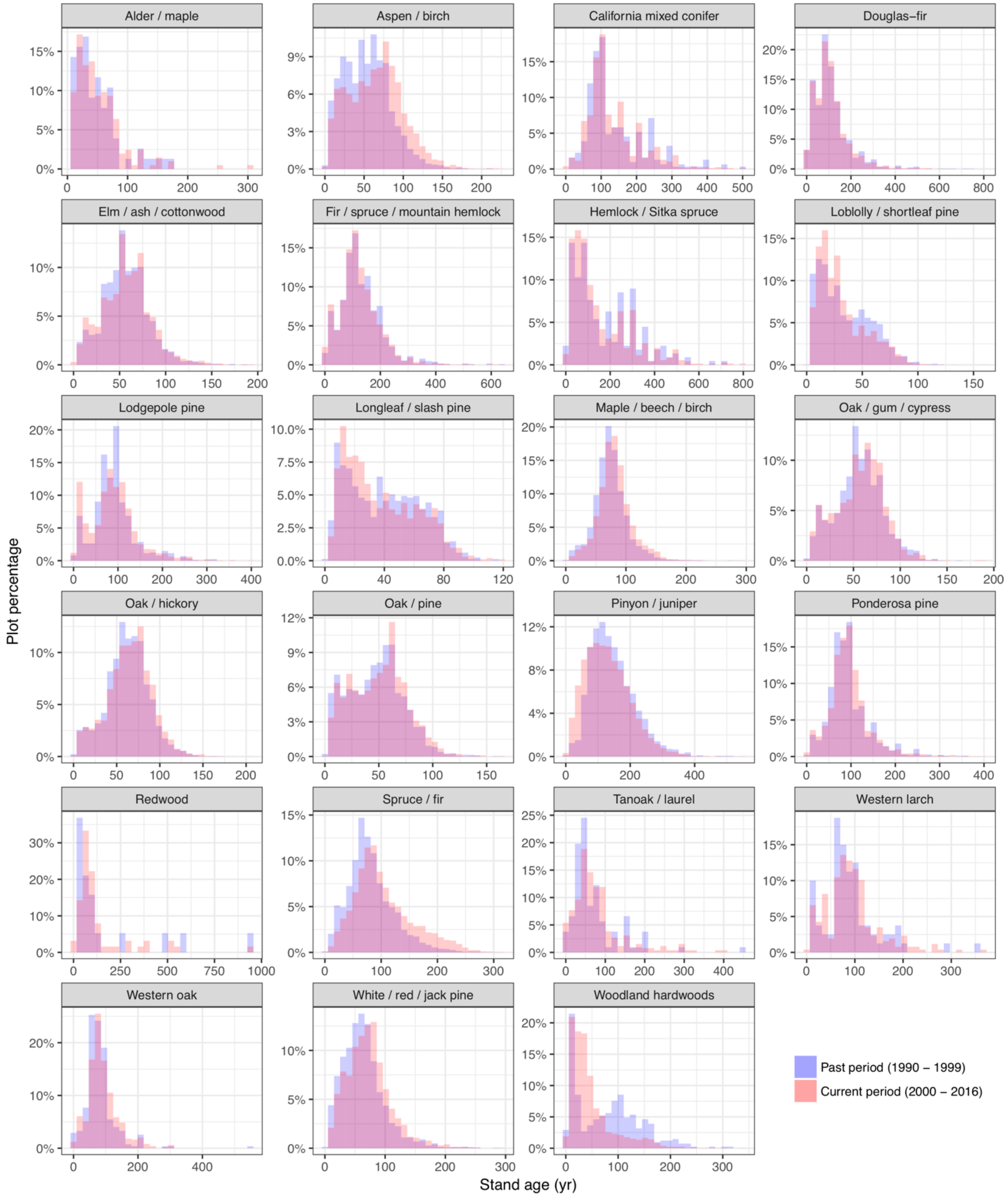

Supplementary Figure 3 Stand age distributions of past and current data. Distributions of plot percentage in 23 primary forest types from the censuses in the past (1990 – 1999) and current (2000 – 2016) periods.

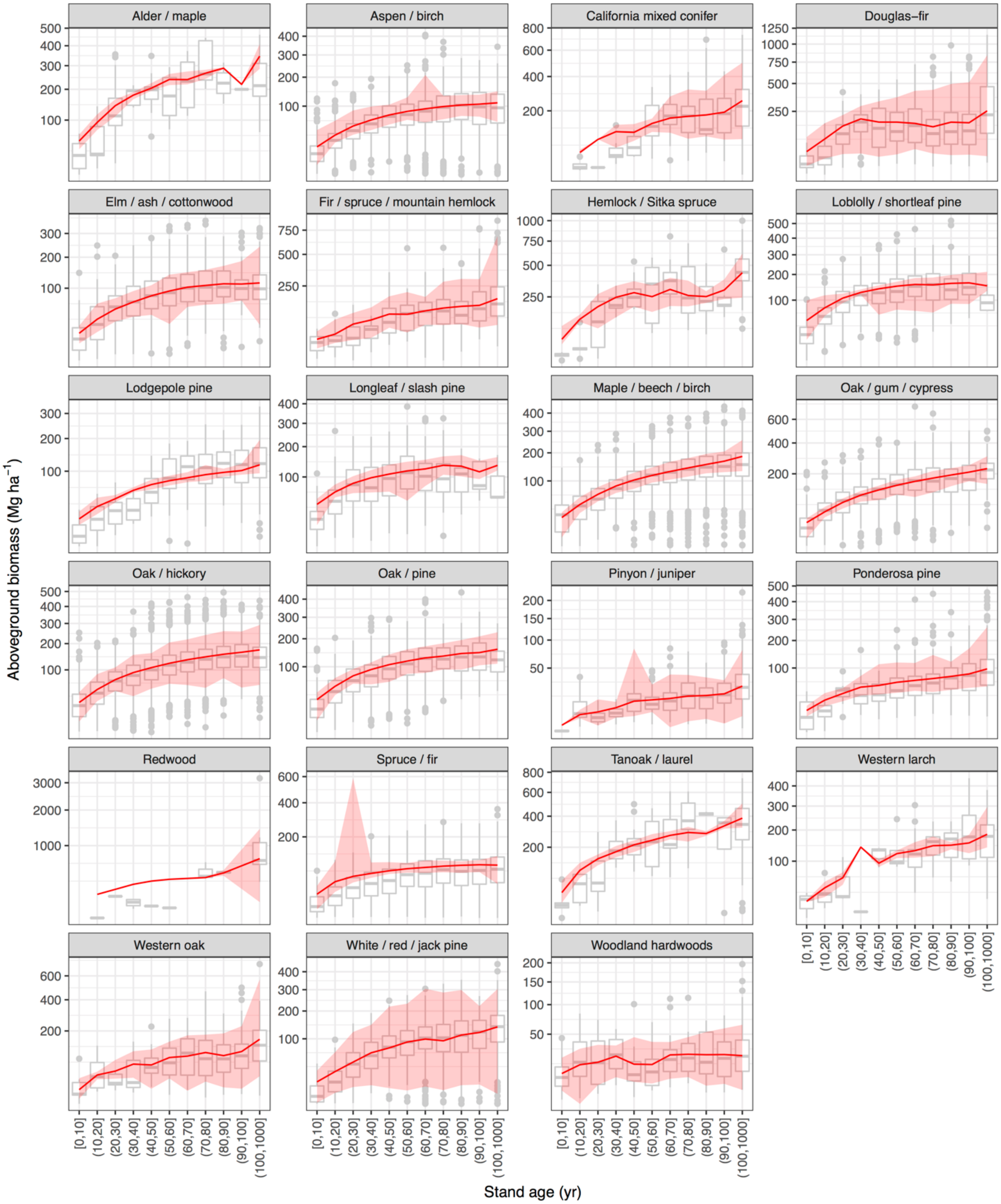

Supplementary Figure 4 Forest aboveground biomass past recovery with stand age across forest-type. Observed and modeled aboveground biomass and stand age in 23 primary forest types across North America are summarized for the past period, 1990 – 1999. Symbols follow Figure 1, except that the modeled values are independent hindcast of the past observation using the current model.

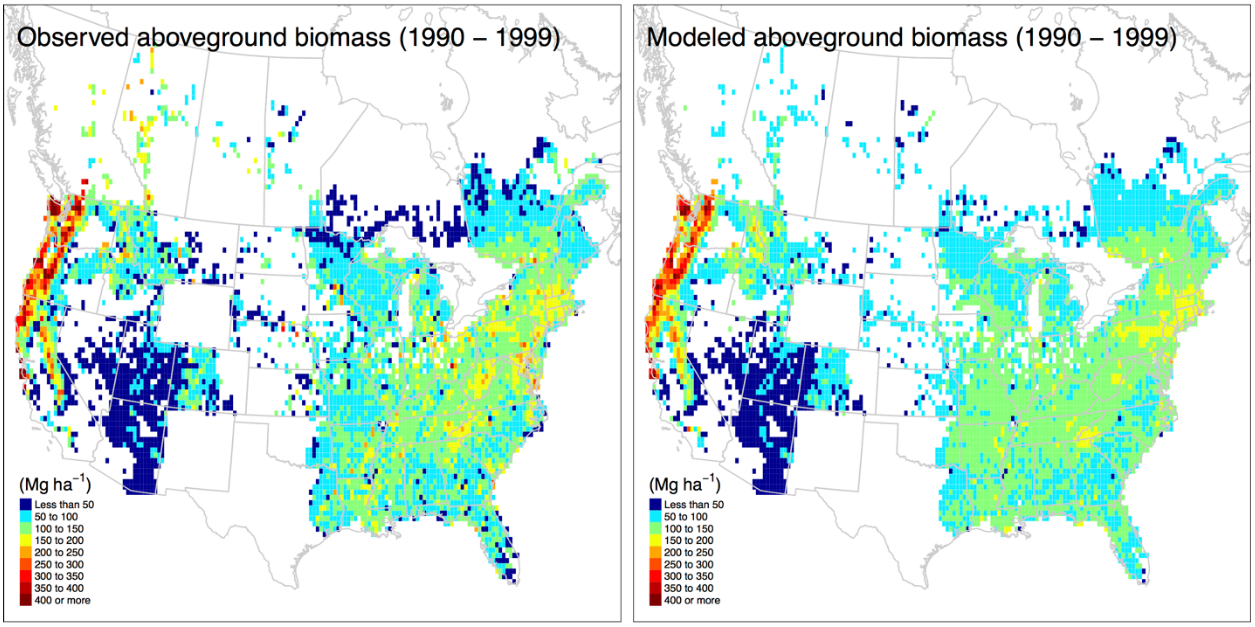

Supplementary Figure 5 Geographic distributions of past forest aboveground biomass. Both the observed and modeled maps are averaged at the 10-min longitude by latitude resolution from inventory plots for the past period, 1990 – 1999. Symbols follow Figure 2, except that the modeled maps are independent hindcast of the past observation using the current model. GIS data source: GADM database of Global Administrative Areas (https://gadm.org).

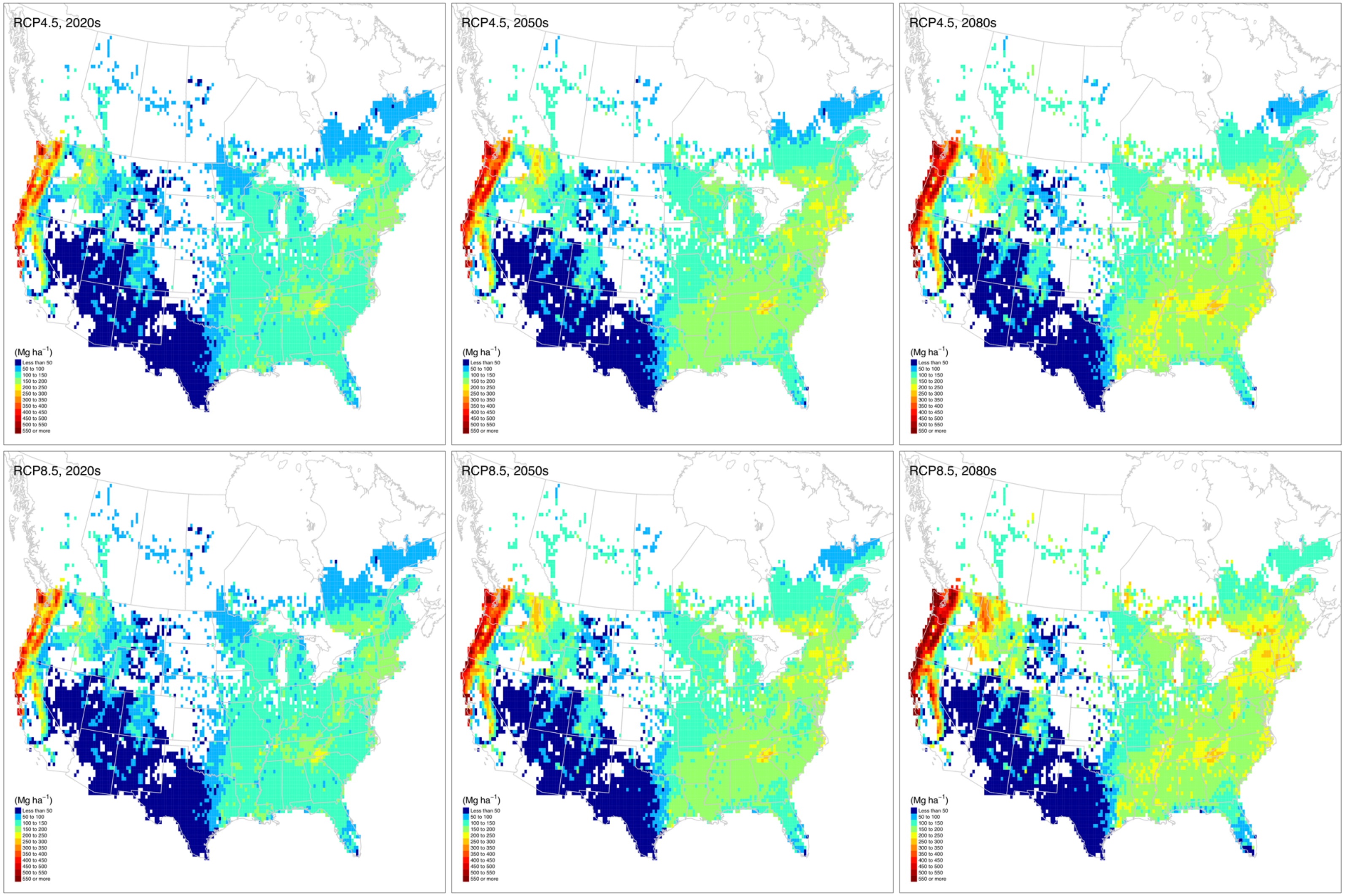

Supplementary Figure 6 Geographic distributions of future forest aboveground biomass. All the modeled maps are summarized for the future periods (2020s, 2050s, 2080s) under emission scenarios (RCP4.5, RCP8.5). Symbols follow Figure 2. GIS data source: GADM database of Global Administrative Areas (https://gadm.org).

Supplementary Table 1 Parameter estimates from current model. For the asymptotic saturated aboveground biomass, the intercept quantifies the saturated biomass under average climate, the temperature effect quantifies the saturated biomass change per 1 °C change in temperature, and the precipitation effect quantifies the saturated biomass change per 1 mm change in precipitation for each forest-type. For the half-saturation stand age, the intercept quantifies the half-saturation age under average climate, the temperature effect quantifies the half-saturation age change per 1 °C change in temperature, and the precipitation effect quantifies the half-saturation age change per 1 mm change in precipitation for each forest-type.

Forest-type	Asymptotic saturated aboveground biomass			Half–saturation stand age		
	Intercept (Mg ha^{-1})	Temperature effect (Mg ha^{-1} °C^{-1})	Precipitation effect (Mg ha^{-1} mm^{-1})	Intercept (yr)	Temperature effect (yr °C^{-1})	Precipitation effect (yr mm^{-1})
Alder / maple	520 (408, 732)	22.6 (–33.8, 65.7)	0.206 (0.059, 0.42)	74.9 (46.5, 131)	–0.102 (–16.8, 12.4)	0.0361 (0.00000487, 0.0867)
Aspen / birch	184 (170, 200)	18.1 (13.9, 22.3)	–0.0228 (–0.0502, 0.00841)	66.1 (56.2, 78.2)	9.35 (6.26, 12)	–0.056 (–0.072, –0.0358)
California mixed conifer	510 (437, 607)	18 (0.793, 36.1)	0.264 (0.147, 0.409)	158 (114, 221)	–16.5 (–27.1, –7.33)	0.0649 (0.00822, 0.136)
Douglas–fir	406 (392, 422)	17.8 (13.9, 21.6)	0.365 (0.344, 0.388)	69.3 (62.4, 77.1)	–6.55 (–8.18, –5)	0.047 (0.041, 0.0531)
Elm / ash / cottonwood	497 (353, 760)	37.4 (18.8, 64.4)	0.0516 (–0.109, 0.223)	228 (146, 385)	18.4 (9.36, 31.8)	–0.0925 (–0.227, 0.00865)
Fir / spruce / mountain hemlock	341 (318, 368)	17.7 (12.4, 23.2)	0.208 (0.177, 0.243)	158 (137, 182)	–13.5 (–17.6, –9.43)	0.0439 (0.026, 0.0625)
Hemlock / Sitka spruce	559 (520, 605)	–6 (–23.3, 11.9)	0.0409 (0.0015, 0.0797)	73 (57.9, 91.8)	–14.9 (–21.3, –8.59)	–0.00577 (–0.0175, 0.00542)
Loblolly / shortleaf pine	249 (237, 261)	22 (18.3, 25.1)	–0.0218 (–0.0818, 0.0456)	33.8 (30.8, 37.2)	5.94 (4.83, 6.93)	–0.00768 (–0.0249, 0.0111)
Lodgepole pine	184 (170, 202)	6.98 (–0.285, 14.8)	0.0901 (0.0265, 0.163)	76.6 (63.6, 93.8)	9.3 (3.04, 16.2)	0.0475 (–0.00426, 0.11)
Longleaf / slash pine	165 (149, 184)	–20.8 (–28.7, –10.9)	–0.113 (–0.218, –0.0133)	23.8 (18.3, 31.2)	–4.38 (–7.18, –0.952)	–0.0252 (–0.0631, 0.0141)
Maple / beech / birch	429 (392, 470)	4.06 (–7.35, 15.8)	0.355 (0.185, 0.536)	148 (129, 170)	–1.24 (–6.71, 4.75)	0.128 (0.0388, 0.224)
Oak / gum / cypress	598 (465, 841)	–65.2 (–105, –24.9)	–0.17 (–0.808, 1.19)	178 (124, 279)	–20.2 (–35.2, –4.94)	–0.0589 (–0.322, 0.431)
Oak / hickory	269 (258, 282)	–11 (–12.6, –9.43)	0.333 (0.305, 0.362)	71.1 (64.9, 78)	–0.909 (–1.8, –0.0059)	0.0465 (0.0346, 0.0592)
Oak / pine	236 (216, 263)	–5.56 (–11.3, 0.848)	0.266 (0.133, 0.387)	55.1 (45.5, 67.7)	–1.82 (–4.61, 1.31)	0.0643 (0.00428, 0.119)
Pinyon / juniper	66.6 (61.8, 71.8)	–6.89 (–7.67, –6.14)	0.113 (0.0974, 0.13)	226 (198, 255)	–9.6 (–12.5, –6.74)	–0.221 (–0.281, –0.16)
Ponderosa pine	153 (139, 170)	–9.35 (–13.2, –4.19)	0.292 (0.246, 0.347)	79.7 (64.5, 101)	–11.3 (–15.9, –5.47)	0.124 (0.0886, 0.169)
Redwood	982 (908, 999)	144 (–79, 436)	1.57 (0.779, 2.39)	86.6 (58.4, 128)	16.2 (–25.3, 84)	0.198 (0.0416, 0.41)
Spruce / fir	112 (107, 118)	12.2 (10.6, 14)	–0.0123 (–0.0215, –0.0027)	27.3 (22.6, 33.2)	9.5 (7.77, 11.2)	–0.026 (–0.0307, –0.0206)
Tanoak / laurel	558 (465, 685)	70 (12.4, 121)	0.0867 (–0.0359, 0.223)	78.9 (52.2, 119)	6.51 (–13.6, 21.8)	0.00322 (–0.0339, 0.0432)
Western larch	319 (254, 429)	–20.7 (–75.9, 35.9)	0.175 (–0.0292, 0.451)	103 (61.4, 179)	–22.9 (–56.8, 9.62)	0.00344 (–0.117, 0.156)
Western oak	310 (233, 467)	–2.89 (–20.4, 12.8)	0.353 (0.241, 0.546)	123 (68.9, 236)	0.353 (–15, 13.6)	0.0908 (0.0361, 0.173)
White / red / jack pine	479 (390, 610)	82.6 (65.9, 106)	–0.44 (–0.618, –0.296)	252 (188, 347)	17.4 (12.6, 23.6)	–0.306 (–0.412, –0.219)
Woodland hardwoods	32.8 (30, 36)	–1.37 (–1.67, –1.07)	0.083 (0.0708, 0.0962)	48.5 (40.1, 58.3)	3.14 (2.34, 3.99)	0.0569 (0.0346, 0.079)
